# Social support and cognitive activity and their associations with incident cognitive impairment in cognitively normal older adults

**DOI:** 10.1186/s12877-024-04655-5

**Published:** 2024-01-09

**Authors:** Tianpei Ma, Jiaqiang Liao, Yuguo Ye, Jiayuan Li

**Affiliations:** 1 Department of Epidemiology and Health Statistics, West China School of Public Health and West China Fourth Hospital, Sichuan University, Chengdu, Sichuan China; 2 Faculty of Public Health & Policy, London School of Hygiene & Tropical Medicine, London, UK

**Keywords:** Cognitive impairment, Social support, Cognitive activity, Health aging, Mediation

## Abstract

**Objectives:**

To explore the associations of social support, and cognitive activity with cognitive impairment incidence, and further examine the mediation effect of cognitive activity on the association between social support and cognitive impairment incidence based on a nationwide elderly Chinese cohort.

**Methods:**

We collected the participants from an ongoing cohort of the Chinese Longitudinal Healthy Longevity Survey (CLHLS). A total of 9394 older adults aged 65 or more years and free of cognitive impairment who participated in the CLHLS between 2008 and 2018 were included. The information on social support and cognitive activity was collected through a questionnaire. The incident cognitive impairment cases were identified through the Mini-Mental State Examination scale (MMSE). Cox proportional hazard regression models were conducted to calculate the hazard ratios (HRs) and 95% confidence interval (CI) of social support and cognitive activity associated with cognitive impairment. We used casual mediation models to assess the indirect association of cognitive activities underlying the association between social support and cognitive impairment.

**Results:**

The adjusted HRs (95% CI) of incident cognitive impairment were 0.956 (0.932 to 0.980), and 0.895 (0.859 to 0.933) associated with per 1 score increase in social support and cognitive activity score, respectively. Better adherence to social support was associated with a higher cognitive activity score (adjusted *β* = 0.046, 95% CI[0.032–0.060]). The baseline cognitive activity, as well as the mean cognitive activity at baseline and during the first follow-up wave, mediate the association between social support and the incidence of cognitive impairment, accounting for 11.4% and 12.6% of the total association, respectively. The participants who were aged 80 years or older, or those with mild daily functional limitations gained more benefits in the development of cognitive activity related to social support, leading to a reduction in the risks of cognitive impairment.

**Conclusion:**

The results of this nationwide cohort provide consistent evidence linking social support, and cognitive activity to reduced risk of subsequent cognitive impairment incidence. These findings provide additional evidence to inform the social strategies to prevent cognitive impairment incidence in elderly people.

**Supplementary Information:**

The online version contains supplementary material available at 10.1186/s12877-024-04655-5.

## Background

The elderly are suffering increased risks of cognitive impairment, which usually involves the decline in one or more cognitive domains, and finally progresses to severe cognitive impairment and dementia [1]. The disease burden related to cognitive impairment is dramatically increasing with the rapid progression of global aging [2]. The number of people living with dementia globally is expected to rise from over 50 million in 2019 to 152 million by 2050, with the annual cost of dementia estimated at USD $1 trillion, set to double by 2030 [3]. Unfortunately, there is currently no effective treatment for dementia, making it crucial to identify modifiable risk factors to reduce or delay cognitive impairment in older adults [4].

Social support is a modifiable contextual factor that was found to help preserve cognitive functioning [5], which refers to a person’s perception of the availability of help or support from others in their social network [6]. The evidence has shown that individuals with less social support are at a higher risk of cognitive impairment, including global cognition and episodic memory [7, 8]. Although several studies have highlighted the mediation roles of emotional encouragement, stress buffering, or engagement in interpersonal activities, the pathway underlying the association between social support on the cognitive function in older adults remains largely unknown [9].

Increasing evidence indicates that physical activity could potentially serve as a link between social support and cognitive function. The findings suggest that there is a positive influence between social support and physical activity levels in older adults [10]. The impact of social support on physical activity may be achieved through encouragement, emotional affirmation, or financial support, or it may be achieved through the transfer of valuable knowledge [11]. Furthermore, physical activity is increasingly documented as a protective factor associated with a lower incidence of cognitive impairment and dementia, providing benefits to adults and younger older adults [12, 13]. However, the evidence regarding these associations among the oldest-old (aged 80 or older) remains limited. Limited by aging and frailty, the improvement of Vigorous- or moderate-intensity outdoor physical activity by social support might be less pronounced among the oldest-old [14].

Fortunately, recent evidence has increasingly highlighted the importance of cognitive activity in promoting cognitive health in older adults [15]. These cognitive activities, which typically involve lighter-intensity activities such as reading books, playing cards, and listening to the radio, have shown better adherence and benefits to cognitive health in older adults [16]. Some studies suggest that cognitive activity has a protective effect on the structure and function of cardiopulmonary and neuromuscular systems [17]. Further evidence has reported similar health benefit pathways of cognitive activity related to physical activity [18], which highlighted the odds of the mediation role of cognitive activity on the association between social support and cognitive functions. However, no studies have been designed to immediately examine these mediation associations, which limited the applications of treating social support as an intervention to improving cognitive function in older adults, especially in those with lower odds of participating in outdoor physical activities.

Therefore, to fill the blank of evidence, we aimed to examine the associations of social support and cognitive activity with subsequent cognitive impairment incidence based on an ongoing nationwide cohort of the Chinese Longitudinal Healthy Longevity Survey (CLHLS). We further examined the mediation role of cognitive activity underlying the association between social support and cognitive impairment incidence.

## Methods

### Study design and participants

The study used data from CLHLS conducted by Peking University’s Research Center for Aging Health and Development. The project has conducted eight follow-up surveys from 1998 to 2018 and has interviewed 113,000 people, covering information on the health status, quality of life, care, and medical needs of the elderly. The sample came from randomly half of the cities and counties in 23 provinces of China since 1998. More information about the cohort can be found in other literature [19, 20].

**Fig. 1:**
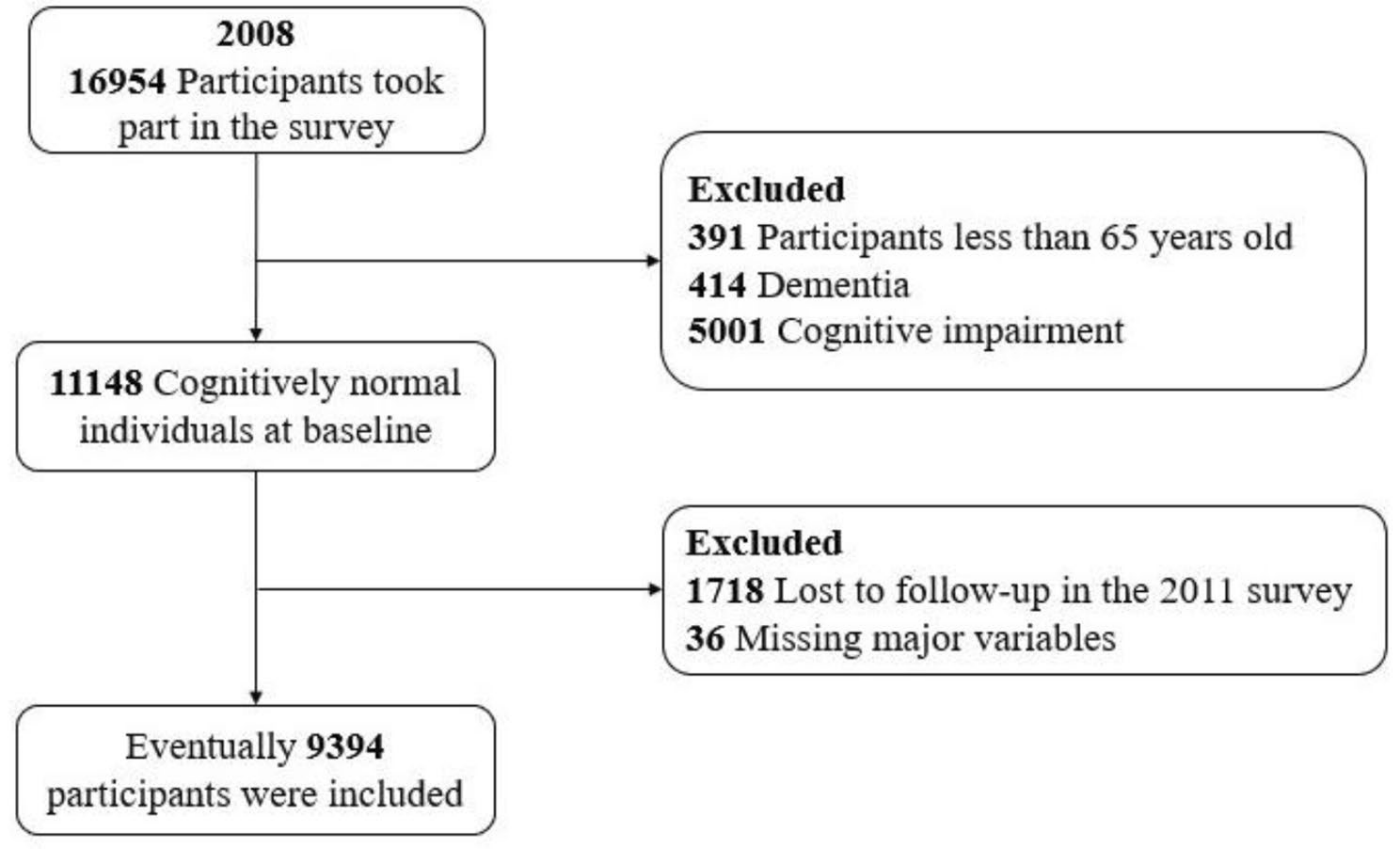
Flow chart of study participants

Our study used CLHLS’s datasets from 2008 to 2018, which included 16,954 participants who were followed up four times in 2008, 2011, 2014, and 2018. We excluded 391 (2.3%) adults under the age of 65 and 5415 (31.9%) participants with dementia or cognitive impairment at baseline. Of the remaining participants, we excluded another 1718 who were lost to follow-up in 2011 and 36 who were missing major variables, resulting in a final analysis population of 9394 participants with complete information on the study variable. The flow chart of research object screening is shown in Fig. 1.

### Exposure: social support

In our study, social support consisted of the variables related to a person’s perception of the availability of emotional help, informational support, or instrumental support from others, divided into support from family and friends, and support from outside the family [21]. Social support from family and friends included contacts with family members and friends (who do you chat with the most; if you have something on your mind or an idea, who do you talk to first; who do you ask first for help when you have problems or difficulties), sick care (whether family members provided care when participants were in sick), money received (whether participants received money from children). Social support outside the family included the perceived availability of social services from the community and social insurance [22]. The total score ranged from 0 to 15, with a higher score indicating a higher level of social support. Specific scoring methods are given in eTable 1.

### Measurements of cognitive activity

Epidemiologic research has shown that a wide range of specific activities can be protective against cognitive decline and Alzheimer’s disease. These activities usually involve significant effort in processing new information, such as crossword puzzles and games [23]. In all studies, leisure activities that require information-seeking and processing are considered “cognitive”. Most studies have focused on a narrow range of leisure activities, including reading books, newspapers, or magazines, doing crosswords or playing cards, and watching television or listening to the radio. However, a few studies have also explored other activities such as participating in group discussions and attending cultural and social events [15].

We used four variables to measure cognitive activity: reading/surfing the Internet, playing cards/mahjong, watching TV/listening to the radio, and participating in social activities. We used a five-point category scale for each item, with 1 being almost every day, and 5 being not attending. We then converted each entry into three categories, 0 = never, 1 = sometimes, 2 = usually, and added all the entries to obtain the total score. The total score of cognitive activity ranged from 0 to 8, with a higher score indicating a higher level of cognitive activity.

### Cognitive impairment assessment

Cognitive function was measured using the Chinese version of the Mini-Mental State Examination scale (MMSE), which included time localization ability, place localization ability, reaction ability, attention and calculation ability, repetition ability, recall ability, naming ability, understanding and self-coordination ability, and visuospatial ability. Getting all 24 questions correctly earns a total of 30 points, with higher scores representing better cognitive function. This scale has been widely used in epidemiological studies of cognitive function screening and has good applicability in the Chinese population [24, 25]. The outcomes were divided according to education level: those without education who scored less than 18 points on the MMSE scale had cognitive impairment; those who have received 1–6 years of education and scored less than 21 points on the MMSE scale were considered to have cognitive impairment; those with more than 6 years of education who scored less than 25 points on the MMSE scale were considered to have cognitive impairment; other conditions were considered normal cognitive function [26, 27].

The time of the first appearance of cognitive impairment after baseline or death, loss of follow-up, and the end of follow-up was recorded as the outcome time, whichever came first. In particular, previous research showed that participants were more likely to be unable to answer relatively difficult tasks when they exhibited poor health and/or existing cognitive limitations. Therefore, following prior research, we categorized responses of “unable to answer” as incorrect answers. This approach has been widely used in previous studies and will not introduce potential bias [28].

### Confounders

The confounders of this study included age, sex (male/female), education (less than primary school/primary school/junior high school and over), residence (rural/town/city), main occupation before age 60 (manual laborers [commercial, service or industrial worker/ self-employed/ agriculture, forestry, animal husbandry or fishery worker/ houseworker/ never worked] or non-manual laborers [professional and technical personnel/ governmental, institutional or managerial personnel/ military personnel]), household income (family’s annual income from last year, less than 12,000 RMB/12,000 RMB or more[divided by the median household income]), living pattern (living with family members/alone/at the nursing home), smoking (no/yes), drinking (no/yes), physical activity score, diet score, activities of daily living (ADL), and the number of chronic diseases. Physical activity score was measured by housework, gardening, personal outdoor activities, and raising domestic animals. The total score of physical activity ranged from 0 to 8, with a higher score indicating a higher level of physical activity. Diet score was calculated according to a previous report [29]. The total score ranged from 0 to 12, and higher scores indicated better adherence to the cMIND (Chinese version of the Mediterranean-DASH Intervention for Neurodegenerative Delay) diet. ADLs were measured by 14 items: dressing, eating, bathing, indoor movement, control of defecation, toilet, visiting neighbor’s house alone, going out shopping alone, cooking, washing clothes, walking 1 km continuously, lifting about 5 kg of heavy objects, squatting and standing up 3 times in succession, and traveling alone by public transportation. For each activity, participants were given one point if they could easily complete it without any help; if they could complete it, but there were certain difficulties or they needed other people to help them complete a specific action, they would get two points; if the participants were unable to complete the activities, they received three points. Then, we added all the items to obtain the total score for follow-up analysis. 14 points represented no ADL limitation. A higher ADL score represented a worse ability to perform daily activities. The categorized ADL variable used the tertiles (the 33% and 66% quartiles) of ADL as the cut-off values.

### Statistical analysis

In the descriptive analyses of the baseline characteristics of the participants, the distributions of continuous variables are summarized by mean (standard deviation), and the categorical variables are described by frequency (percentage). We used the Chi-square test (categorical variable), or ANOVA analysis (continuous variable) to infer the significance of baseline difference. The Cox proportional hazard regression model was used to assess the associations of social support and cognitive activity with subsequent risk of incident cognitive impairment. We performed the Schoenfeld residual error tests to examine the assumption of proportional hazards assumptions in Cox regression models. A generalized linear model was used to assess the linear trend of the social support and cognitive activity tertiles (the 33% and 66% quartiles), respectively, treating the tertiles as continuous variables (1–3) and conducting linear correlation analysis with cognitive impairment.

**Fig. 2:**
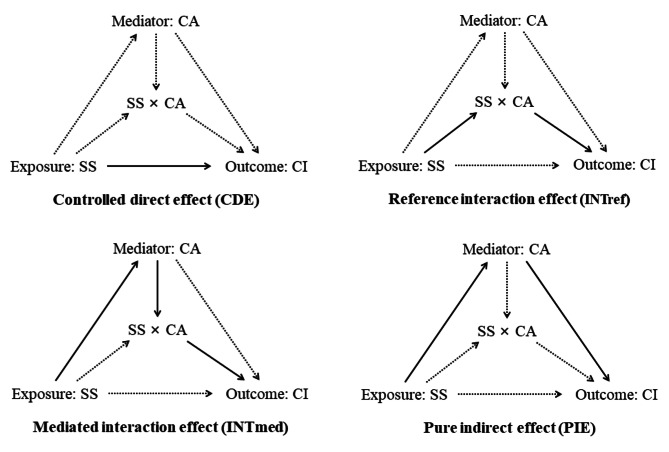
Conceptual framework of the research using four-way decomposition mediation analysis. Abbreviations: SS = Social support; CA = Cognitive activity; CI = Cognitive impairment. Note: A solid line represents a practical path, while a dashed line indicates that the path is blocked

For assessing mediations of the social support on cognitive impairment incidence through developing cognitive activity, we applied the 2-stage regression method for survival data proposed by VanderWeele [30]. We specified a linear regression model for the mediator (cognitive activity) adjusted for social support and confounders and a Cox proportional hazards regression model for the outcome (cognitive impairment) adjusted for social support, cognitive activity, and confounders. The following potential confounders were included in both models: age, sex, education, residence, occupation, household income, living pattern, smoking, drinking, physical activity score, diet score, ADL, and number of chronic diseases. We used the Med4way [31, 32] command to calculate the mediating and interacting effects in this study. Med4way can be used when the outcome is continuous, dichotomous, count, or survival time, and the mediator is continuous or binary. This method assumed that the total effect (TE) of social support on cognitive impairment can decompose into four components: the controlled direct effect (CDE, due neither to mediation nor interaction), the reference interaction (INTref, due to interaction only), the mediated interaction (INTmed, due to mediation and interaction) and the pure indirect effect (PIE, due to mediation only). The estimates of the total effect and four components were presented as excess risk ratio (RR), i.e., RR minus (1) The specific paths of the four-way effect decomposition and explanation were shown in Fig. 2 and eTable (2) Then, we used age, sex, education, household income, and ADL as grouping variables for subgroup analysis.

Finally, we conducted analogous mediation analyses excluding participants who died in 2011, using individual chronic diseases as confounders, using cognitive activity without participating in social activities as a mediator, and focusing study population aged 60 years and older for sensitivity analyses.

All statistical tests were 2-sided at a significance level of *P* < 0.05. Mediation analysis was performed using the “Med4way” command in Stata 15.0 [31]. Another statistical analysis was conducted in R, version 4.2.2.

## Result

### Demographic characteristics

The analyzed cohort included 9394 participants (4570 men [48.6%],4824 women [51.4%] initially free of cognitive impairment, with an average age of 83.54 (10.78), ranging from 65 to 116 years old. Among them, 17.8%(*n* = 1674)developed cognitive impairment during a mean follow-up of 5.1 (3.2) years (47,697 person-years of follow-up). The mean MMSE score at baseline was 26.85 (3.29). The mean cognitive activity score and social support score at baseline were 2.10 (1.63) and 10.94 (2.23), respectively. Cognitive impairment was associated with lower levels of social support and cognitive activity, but also presented in females, older individuals, lower education, manual laborers, poor marital status, smoking, drinking, lower physical activity score and diet score, and worse ability of daily living (Table 1).

**Table 1:** The distributions of baseline characteristics of the study participants stratified by cognitive status

Variables		Overall	No cognitive impairment	Cognitive impairment	*P*-value
N (%)		9394	7720(82.2%)	1674(17.8%)	
Sex (%)	Male	4570 (48.6)	3942 (51.1)	628 (37.5)	< 0.001
	Female	4824 (51.4)	3778 (48.9)	1046 (62.5)	
Baseline age (mean [SD])		83.54 (10.78)	83.00 (10.96)	86.06 (9.50)	< 0.001
Education (%)	Less than primary school	5355 (57.0)	4259 (55.2)	1096 (65.5)	< 0.001
	Primary school	2992 (31.9)	2571 (33.3)	421 (25.1)	
	Junior high school and over	1047 (11.1)	890 (11.5)	157 (9.4)	
Occupation (%)^a^	Manual laborers	8566 (91.2)	6995 (90.6)	1571 (93.8)	< 0.001
	Non-manual laborers	828 (8.8)	725 (9.4)	103 (6.2)	
Household income (%)^b^	Less than 12,000 CNY	4795 (51.0)	3927 (50.9)	868 (51.9)	0.482
	12,000 CNY or more	4599 (49.0)	3793 (49.1)	806 (48.1)	
Residence (%)	Urban	1617 (17.2)	1323 (17.1)	294 (17.6)	0.200
	Town	1947 (20.7)	1627 (21.1)	320 (19.1)	
	Rural	5830 (62.1)	4770 (61.8)	1060 (63.3)	
Marital status (%)	Married	3879 (41.3)	3363 (43.6)	516 (30.8)	< 0.001
	Divorced/ widowed/ never married	5515 (58.7)	4357 (56.4)	1158 (69.2)	
Living with (%)	Living with family members	7739 (82.4)	6383 (82.7)	1356 (81.0)	0.202
	Alone	1535 (16.3)	1243 (16.1)	292 (17.4)	
	At nursing home	120 (1.3)	94 (1.2)	26 (1.6)	
Smoking (%)	No	7451 (79.3)	6068 (78.6)	1383 (82.6)	< 0.001
	Yes	1943 (20.7)	1652 (21.4)	291 (17.4)	
Drinking (%)	No	7524 (80.1)	6132 (79.4)	1392 (83.2)	0.001
	Yes	1870 (19.9)	1588 (20.6)	282 (16.8)	
Physical activity score (mean [SD])		3.15 (2.02)	3.19 (2.03)	2.97 (2.00)	< 0.001
Diet score (mean [SD])		4.88 (1.58)	4.91 (1.59)	4.75 (1.52)	< 0.001
ADLs (mean [SD])		17.95 (5.57)	17.79 (5.54)	18.67 (5.64)	< 0.001
Chronic disease No. (mean [SD])		0.98 (1.17)	0.98 (1.18)	0.98 (1.14)	0.968
Cognitive activity score at baseline (mean [SD])		2.10 (1.63)	2.16 (1.64)	1.80 (1.55)	< 0.001
Social support score at baseline (mean [SD])		10.94 (2.23)	11.02 (2.24)	10.56 (2.17)	< 0.001
MMSE score at baseline (mean [SD])		26.85 (3.29)	26.99 (3.23)	26.19 (3.44)	< 0.001

Note: a: manual laborers including commercial, service or industrial workers/ self-employed/ agriculture, forestry, animal husbandry or fishery worker/ houseworker/ never worked; non-manual laborers including professional and technical personnel/ governmental, institutional or managerial personnel/ military personnel. b: The median of household income (12,000 CNY, or about 1700 USD) was taken as the cut-off value

### The risk of cognitive impairment by social support and cognitive activity

In this study, better baseline adherence to social support and cognitive activity were associated with lower risks of subsequent incident cognitive impairment (Table 2). The multivariable-adjusted HRs and 95% confidence interval (CI) were 0.956 (0.932 to 0.980), and 0.895 (0.859 to 0.933) associated with a per 1 score increase in social support and cognitive activity score, respectively. Similar associations were obtained by replacing the measurements of social support and cognitive activity by tertiles.

**Table 2:** The risk of cognitive impairment by social support and cognitive activity

	Social support, HR(95%CI for HR)			Cognitive activity, HR(95%CI for HR)	
	Unadjusted	Adjusted		Unadjusted	Adjusted
**Scores(continuous)**	0.867(0.851,0.883)**	0.956(0.932,0.980)**		0.757(0.732,0.784)**	0.895(0.859,0.933)**
**Status by tertiles**					
T1	1[Reference]	1[Reference]		1[Reference]	1[Reference]
T2	0.803(0.698,0.925)**	0.898(0.779,1.036)		0.532(0.475,0.596)**	0.755(0.671,0.850)**
T3	0.400(0.357,0.448)**	0.794(0.692,0.912)**		0.390(0.346,0.440)**	0.712(0.614,0.825)**
*P* for trend	< 0.001**	0.071*		< 0.001**	0.026**

Abbreviations: HR = hazard ratio; T = tertiles

Note: The following adjusted variables were included in each model: age, sex, education, residence, occupation, household income, living pattern, smoking, drinking, physical activity score, diet score, ADL, and number of chronic diseases. *P* for trend, obtained from the Generalized Linear Model of a linear association of the tertiles as continuous variables (1–3) with risk of cognitive impairment. * *P* < 0.1, ** *P* < 0.05

### The association between social support and cognitive activity

There was a positive association between social support and cognitive activity. Better adherence to social support was associated with greater odds of participating in cognitive activity (adjusted *β* = 0.046, 95% CI[0.032–0.060]) (Table 3).

**Table 3:** Linear regression coefficients (95%CI) for the association between social support and cognitive activity

	Coefficient per point of the cognitive activity	95% CI		*P*-value
Model 1	0.205	0.191	0.220	< 0.001
Model 2	0.046	0.032	0.060	< 0.001

Note: Model 1 adjusted social support only. Model 2: Model 1 + age, sex, education, residence, occupation, household income, living pattern, smoking, drinking, physical activity score, diet score, ADL, and number of chronic diseases

### The effects of social support on cognitive impairment due to mediation and interaction with cognitive activity

**Fig. 3:**
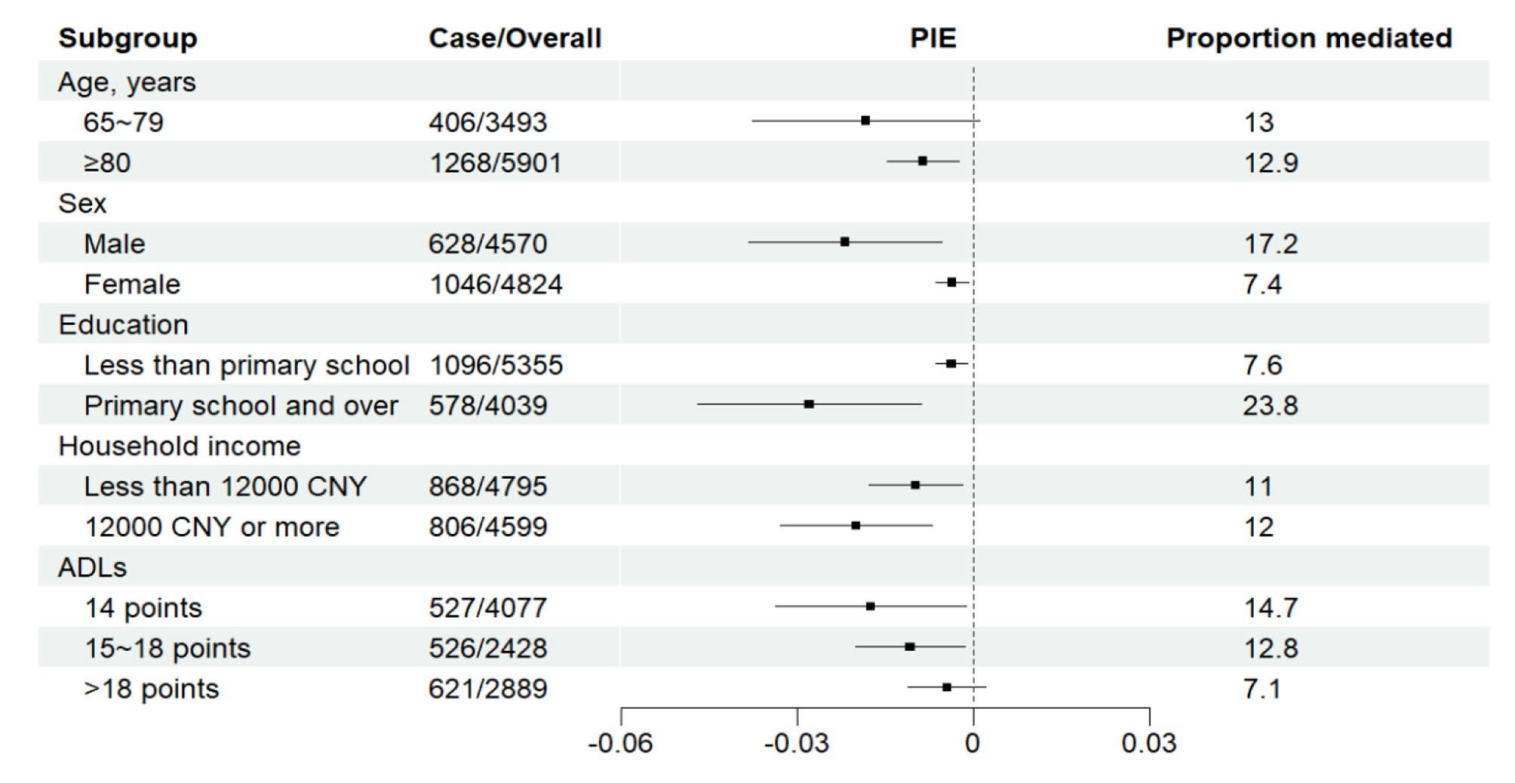
Subgroup analysis of mediating effects of cognitive activities. Abbreviations: PIE = pure indirect effect. Note: The tertiles of ADLs score were taken as the cut-off value. The forest plot represents the 95% confidence interval for Excess RR of PIE

We explored the mediating and interacting role of cognitive activity in the effect of social support on cognitive impairment in the elderly. For all analyses, the cognitive activity was treated as a mediator, and the total effect of social support on cognitive impairment was significant (excess risk ratio [RR] = -0.125, 95%CI[-0.193,-0.057]) which can be divided into four parts. The controlled direct effect (excess RR=-0.112, 95%CI[-0.180,-0.043]) of social support on cognitive impairment, and pure mediation effect of cognitive activity as a mediator (excess RR=-0.014, 95%CI[-0.022,-0.007]) were significant. However, the effect of reference interaction and mediated interaction were not significant (*P* > 0.05). The proportion of the controlled direct effect and pure indirect effect were 89.4% and 11.4%, respectively. Similarly, replacing the mediator of baseline cognitive activity by the mean of cognitive activity scores between 2008 and 2011, the direct effect and indirect effect were − 0.118(95%CI[-0.233, -0.004]) and − 0.017 (95%CI[-0.028, -0.006]), respectively, and the indirect effect accounted for 12.6% of the total association (Table 4). The results of subgroup analysis showed that the mediating effect of cognitive activity was primarily in individuals who were over 80 years old, individuals with mild daily functional limitations (ADL score ranged from 15 to 18), or individuals with normal daily function (Fig. 3, eTable 3).

**Table 4:** The effects of social support on cognitive impairment due to mediation and interaction with cognitive activity

Variable	The CA score in 2008 as a mediator			The mean of CA score in 2008 and 2011 as a mediator ^a^	
	Excess RR (95%CI)	% mediated		Excess RR (95%CI)	% mediated
Total effect	-0.125(-0.193,-0.057)*	100.0		-0.137(-0.244,-0.031)*	100.0
CDE	-0.112(-0.180,-0.043)*	89.4		-0.118(-0.233,-0.004)*	85.9
INTref	0.001(-0.004,0.005)	-0.5		0.001(-0.012,0.012)	0.1
INTmed	0.001(-0.006,0.006)	-0.3		-0.002(-0.009,0.005)	1.4
PIE	-0.014(-0.022,-0.007)*	11.4		-0.017(-0.028,-0.006)*	12.6

Abbreviations: CDE = controlled direct effect; INTref = reference interaction effect; INTmed = mediated interaction effect; PIE = pure indirect effect; RR = risk ratio; CA = cognitive activity

Note: The following adjusted variables were included in each model: age, sex, education, residence, occupation, household income, living pattern, smoking, drinking, physical activity score, diet score, ADL, and number of chronic diseases. * *P* < 0.05. a, Participants who had cognitive impairment in 2011 and had a missing CA score in 2011 were excluded

After excluding participants who died in 2011, using individual chronic diseases as confounders, using cognitive activity without participating in social activities as a mediator, and focusing study population aged 60 years and older, the results remained little changes in the main results (eTable 4). Overall, the cognitive activity performed significant mediating roles in the relation of social support and cognitive impairment.

## Discussion

In this nationwide prospective cohort, we provided compelling evidence linking higher levels of social support and cognitive activity to a lower subsequent incidence of cognitive impairment in Chinese older adults. Our findings underscore the mediating role of cognitive activity in enhancing the likelihood of mitigating cognitive impairment associated with social support, illuminating a potentially modifiable pathway for intervention. The strength of these associations persisted even after adjustments for potential confounding variables and comprehensive sensitivity analyses. Particularly striking is the pronounced significance of these associations within the oldest-old and those with mild daily functional limitations. These findings emphasize the critical importance of targeting and improving the “social support-cognitive activity” pathway as a preventive strategy to effectively reduce the risk of cognitive impairment incidence among both the oldest-old population and older adults with mild daily functional limitations.

After adjusting for other confounders, we observed a significant protective association between social support and cognitive impairment in older adults (HR = 0.956, 95%CI[0.932–0.980]), consistent with prior research in this field [22, 33]. It is plausible that higher levels of perceived social support have a favorable influence on an individual’s lifestyle behaviors, such as seeking medical care, engaging in regular exercise, adhering to medication regimens, and quitting smoking, which collectively contribute to improved cognitive function and overall health outcomes [34]. This protective association could be attributed to the opportunities for communication and interpersonal interaction that social support networks provide, potentially contributing to a reduction in cognitive decline [33]. Additionally, social support has been demonstrated to protect individuals against the adverse consequences of stressful events, thereby fostering positive impacts on well-being and cognition [9]. Despite the protective impact of social support on cognitive function, changes in life circumstances such as retirement, loss of a partner or friend, and changes in financial status may lead to a decrease in the level of available social support among older adults [35]. Therefore, further research into the potential pathways linking social support and cognitive health is crucial for targeted interventions in this population.

The finding related to cognitive activity is supported by prior research showing the protective association of cognitive activity with cognitive impairment in older adults(HR = 0.895, 95%CI[0.859–0.933]) [12, 36]. Mechanisms by how participation in cognitive activity may influence the incidence of cognitive impairment include enhanced cognitive reserve through the use of more effective neural networks [37, 38]. A whitepaper published by Reserve, Resilience, and Protective Factors Professional Interest Area, defines cognitive reserve refers to “the adaptability (i.e., efficiency, capacity, flexibility) of cognitive processes that helps to explain differential susceptibility of cognitive abilities or day-to-day function to brain aging, pathology or insult” [39]. Current evidence suggests cognitive reserve can develop throughout individuals’ lives via experiences like stimulating cognitive activity, education, and work [40]. These experiences can enhance neural connectivity and cognitive abilities, acting as a protective shield against disease-related changes and enabling the brain to compensate for damage by activating alternative neural pathways [40]. In essence, this supports the “use it or lose it” concept, where cognitive engagement strengthens neural circuits, promotes brain plasticity, and optimizes brain networks’ efficiency through activities that encourage neurogenesis and increased synaptic density [41].

We presented prospective population evidence suggesting that cognitive activity mediates the relationship between social support and cognitive impairment with the mediating effect estimated at 11.4%. One plausible explanation was that a supportive environment from families and society could stimulate or encourage healthy behaviors, such as cognitive activity, and provide various sources of information, which could help to have a positive impact on health [42]. Consequently, individuals with higher levels of social support who engaged in more cognitive activity to maintain brain stimulation and release stress exhibited a reduced likelihood of developing cognitive impairment. Specially, when using the mean of cognitive activity scores in 2008 and 2011 as a mediator, the indirect effect increased from 11.4 to 12.6% underscoring the sustained temporal persistence of the mediating effect of cognitive activity.

Several early studies have suggested that social interaction involved in some kinds of activities may be more relevant to cognitive function [43]. However, the results of mediation analyses in this study did not support the significant social support-cognitive activity interactions underlying the association between social support and cognitive impairment incidence in older adults. Additionally, to further confirm the odds of these interactions, we replaced the definitions of cognitive activities by excluding the variables related to social activities and replicated the mediation analyses. The significant evidence indicated the mediation role of cognitive activity on the study associations (eTable4). All of these results consistently supported the mediation roles of reading/surfing the Internet, playing cards/mahjong, and watching TV/listening to the radio on the association between social support and cognitive impairment incidence in older adults.

Significantly, we observed that the oldest-old and older adults with mild daily functional limitations derived greater benefits from the mediating effects discussed earlier. Existing evidence has indicated a higher incidence of cognitive impairment in the oldest-old and older adults with mild daily functional limitations [24, 44]. While the link between exercise and reduced dementia risk is well-established [37], specific exercise recommendations for individuals aged 80 and older remain limited. Vigorous- or moderate-intensity exercise may not be appropriate as an intervention for maintaining and preserving cognitive function in this age group or those with mild daily functional limitations. Exploring strategies for maintaining social support among the very elderly or those with mild daily functional limitations may offer a promising approach to postponing cognitive decline. Therefore, strengthening the positive influence of social support on cognitive impairment in these older adults through cognitive activity presents a highly effective avenue for promoting cognitive health.

As the population continues to age, initiatives aimed at enhancing economic, social, and educational opportunities may exert a profound impact on cognitive and brain health among older adults [45, 46]. For instance, encouraging families to provide increased financial and emotional support to older individuals, promoting a variety of cognitive activities (e.g., chess and card competitions), expanding learning opportunities within older communities (e.g., establishing senior universities and libraries), and fostering social connections (e.g., creating green spaces and senior activity center) may all contribute to the promotion of cognitive health [47].

This study contributes valuable insights to the field of geriatric neurology and highlights the potential for public health interventions aimed at preserving cognitive health in aging populations. However, some limitations should be mentioned in this study. Firstly, the data on social support and cognitive activity relied on self-reporting, which may cause recall bias. Secondly, some unmeasured potential covariates (such as medication use) might confound the association between social support, cognitive activity, and cognitive impairment. Thirdly, comparing the characteristics of participants and those who were lost to follow-up, it was found that residents with higher household incomes and current residence in cities were more likely to be lost to follow-up. Therefore, for older adults with higher socioeconomic status, more caution should be used when interpreting the study results. Finally, cognitive function in this study was assessed only by MMSE, and no clinical assessment or other cognitive testing in subdomains was performed.

## Conclusion

This study provided compelling evidence linking higher levels of social support and cognitive activity to a lower subsequent incidence of cognitive impairment in Chinese older adults. Particularly striking is the pronounced significance of these associations within the oldest-old and those with mild daily functional limitations. As the population continues to age, initiatives to improve economic, social, and educational opportunities may have a profound impact on cognitive and brain health in older adults. It is crucial to provide older individuals with increased mental, emotional, and material support while encouraging their engagement in more cognitive activities during leisure time to mitigate the risk of cognitive impairment.

### Electronic supplementary material

Below is the link to the electronic supplementary material.

Supplementary Material 1

## Data Availability

All data from the CLHLS are publicly available at https://opendata.pku.edu.cn/dataverse/CHADS.

## Abbreviations

CLHLS: Chinese Longitudinal Healthy Longevity Survey
MMSE: Chinese version of the Mini-Mental State Examination scale
ADL: Activities of daily living
cMIND: Chinese version of the Mediterranean-DASH Intervention for Neurodegenerative Delay
HR: Hazard ratio
95%CI: 95% Confidence interval
RR: Risk ratio
CDE: Controlled direct effect
INTref: Reference interaction effect
INTmed: Mediated interaction effect
PIE: Pure indirect effect
SS: Social support
CA: Cognitive activity
CI: Cognitive impairment

## References

[1] Owens DK, Davidson KW, Krist AH, Barry MJ, Cabana M, Caughey AB, Doubeni CA, Epling JW, Kubik M, Landefeld CS (2020). Screening for cognitive impairment in older adults: US Preventive Services Task Force Recommendation Statement. JAMA.

[2] The Lancet Public H (2021). Reinvigorating the public health response to dementia. Lancet Public Health.

[3] International AsD.: World Alzheimer Report 2019: Attitudes to dementia. In.: Alzheimer’s Disease International; 2019.

[4] Elahi FM, Miller BL (2017). A clinicopathological approach to the diagnosis of dementia. Nat Rev Neurol.

[5] Oremus M, Tyas SL, Maxwell CJ, Konnert C, O’Connell ME, Law J (2020). Social support availability is positively associated with memory in persons aged 45–85 years: a cross-sectional analysis of the Canadian longitudinal study on aging. Arch Gerontol Geriatr.

[6] Berkman LF, Glass T, Brissette I, Seeman TE (2000). From social integration to health: Durkheim in the new millennium. Soc Sci Med.

[7] Posis AIB, Yarish NM, McEvoy LK, Jain P, Kroenke CH, Saquib N, Ikramuddin F, Schnatz PF, Bellettiere J, Rapp SR (2023). Association of Social Support with mild cognitive impairment and dementia among older women: the women’s Health Initiative Memory Study. J Alzheimers Dis.

[8] Kelly ME, Duff H, Kelly S, McHugh Power JE, Brennan S, Lawlor BA, Loughrey DG (2017). The impact of social activities, social networks, social support and social relationships on the cognitive functioning of healthy older adults: a systematic review. Syst Rev.

[9] Cohen S (2004). Social relationships and health. Am Psychol.

[10] Lindsay Smith G, Banting L, Eime R, O’Sullivan G, van Uffelen JGZ (2017). The association between social support and physical activity in older adults: a systematic review. Int J Behav Nutr Phys Act.

[11] Newsom JT, Shaw BA, August KJ, Strath SJ (2018). Physical activity-related social control and social support in older adults: cognitive and emotional pathways to physical activity. J Health Psychol.

[12] Najar J, Östling S, Gudmundsson P, Sundh V, Johansson L, Kern S, Guo X, Hällström T, Skoog I (2019). Cognitive and physical activity and dementia: a 44-year longitudinal population study of women. Neurology.

[13] Sofi F, Valecchi D, Bacci D, Abbate R, Gensini GF, Casini A, Macchi C (2011). Physical activity and risk of cognitive decline: a meta-analysis of prospective studies. J Intern Med.

[14] McPhee JS, French DP, Jackson D, Nazroo J, Pendleton N, Degens H (2016). Physical activity in older age: perspectives for healthy ageing and frailty. Biogerontology.

[15] Sajeev G, Weuve J, Jackson JW, VanderWeele TJ, Bennett DA, Grodstein F, Blacker D (2016). Late-life cognitive activity and dementia: a systematic review and Bias Analysis. Epidemiology.

[16] Wilson RS, De Mendes CF, Barnes LL, Schneider JA, Bienias JL, Evans DA, Bennett DA (2002). Participation in cognitively stimulating activities and risk of incident Alzheimer disease. JAMA.

[17] Park DC, Bischof GN (2013). The aging mind: neuroplasticity in response to cognitive training. Dialogues Clin Neurosci.

[18] Landau SM, Marks SM, Mormino EC, Rabinovici GD, Oh H, O’Neil JP, Wilson RS, Jagust WJ (2012). Association of lifetime cognitive engagement and low β-amyloid deposition. Arch Neurol.

[19] Zeng Y, Feng Q, Hesketh T, Christensen K, Vaupel JW (2017). Survival, disabilities in activities of daily living, and physical and cognitive functioning among the oldest-old in China: a cohort study. Lancet.

[20] Lv X, Li W, Ma Y, Chen H, Zeng Y, Yu X, Hofman A, Wang H (2019). Cognitive decline and mortality among community-dwelling Chinese older people. BMC Med.

[21] Tian G, Li R, Cui Y, Zhou T, Shi Y, Yang W, Ma Y, Shuai J, Yan Y (2022). Association between disability, social support and depressive symptoms in Chinese older adults: a national study. Front Public Health.

[22] Yin S, Yang Q, Xiong J, Li T, Zhu X (2020). Social Support and the incidence of cognitive impairment among older adults in China: findings from the Chinese longitudinal healthy longevity Survey Study. Front Psychiatry.

[23] Jonaitis E, La Rue A, Mueller KD, Koscik RL, Hermann B, Sager MA (2013). Cognitive activities and cognitive performance in middle-aged adults at risk for Alzheimer’s disease. Psychol Aging.

[24] Zhang Q, Wu Y, Han T, Liu E. Changes in cognitive function and risk factors for cognitive impairment of the Elderly in China: 2005–2014. Int J Environ Res Public Health 2019, 16(16). 10.3390/ijerph16162847 PMC6719934 31404951

[25] Zhang P-D, Lv Y-B, Li Z-H, Yin Z-X, Li F-R, Wang J-N, Zhang X-R, Zhou J-H, Wu X-B, Duan J (2020). Age, Period, and Cohort effects on activities of Daily Living, Physical Performance, and cognitive functioning impairment among the Oldest-Old in China. J Gerontol A Biol Sci Med Sci.

[26] Cui GH, Yao YH, Xu RF, Tang HD, Jiang GX, Wang Y, Wang G, Chen SD, Cheng Q (2011). Cognitive impairment using education-based cutoff points for CMMSE scores in elderly Chinese people of agricultural and rural Shanghai China. Acta Neurol Scand.

[27] Zhang MY, Katzman R, Salmon D, Jin H, Cai GJ, Wang ZY, Qu GY, Grant I, Yu E, Levy P (1990). The prevalence of dementia and Alzheimer’s disease in Shanghai, China: impact of age, gender, and education. Ann Neurol.

[28] Yang F, Cao J, Qian D, Ma A. Stronger increases in cognitive functions among Socio-economically disadvantaged older adults in China: a longitudinal analysis with multiple birth cohorts. Int J Environ Res Public Health 2020, 17(7). 10.3390/ijerph17072418 PMC7177365 32252350

[29] Huang X, Aihemaitijiang S, Ye C, Halimulati M, Wang R, Zhang Z (2022). Development of the cMIND Diet and its association with cognitive impairment in older Chinese people. J Nutr Health Aging.

[30] VanderWeele TJ (2011). Causal mediation analysis with survival data. Epidemiology.

[31] Discacciati A, Bellavia A, Lee JJ, Mazumdar M, Valeri L. Med4way: a Stata command to investigate mediating and interactive mechanisms using the four-way effect decomposition. Int J Epidemiol 2018:15–20. 10.1093/ije/dyy236 30452641

[32] Yu B, Feng C, Yang X, Wang Z, Zou H, Jia P, Yang S (2023). Roles of Social Capital in the Association between Internalized Homophobia and Condomless Sex among men who have sex with men in Southwest China: a four-way decomposition. Int J Public Health.

[33] Pillemer SC, Holtzer R (2016). The differential relationships of dimensions of perceived social support with cognitive function among older adults. Aging Ment Health.

[34] Seeman TE, Crimmins E. Social environment effects on health and aging: integrating epidemiologic and demographic approaches and perspectives. Ann N Y Acad Sci 2001, 954. 10.1111/j.1749-6632.2001.tb02749.x 11797869

[35] Czaja SJ, Moxley JH, Rogers WA (2021). Social support, isolation, loneliness, and Health among older adults in the PRISM randomized controlled trial. Front Psychol.

[36] Karssemeijer EGA, Aaronson JA, Bossers WJ, Smits T, Olde Rikkert MGM, Kessels RPC (2017). Positive effects of combined cognitive and physical exercise training on cognitive function in older adults with mild cognitive impairment or dementia: a meta-analysis. Ageing Res Rev.

[37] Livingston G, Huntley J, Sommerlad A, Ames D, Ballard C, Banerjee S, Brayne C, Burns A, Cohen-Mansfield J, Cooper C (2020). Dementia prevention, intervention, and care: 2020 report of the Lancet Commission. Lancet.

[38] Leung GTY, Fung AWT, Tam CWC, Lui VWC, Chiu HFK, Chan WM, Lam LCW (2011). Examining the association between late-life leisure activity participation and global cognitive decline in community-dwelling elderly Chinese in Hong Kong. Int J Geriatr Psychiatry.

[39] Stern Y, Arenaza-Urquijo EM, Bartrés-Faz D, Belleville S, Cantilon M, Chetelat G, Ewers M, Franzmeier N, Kempermann G, Kremen WS (2020). Whitepaper: defining and investigating cognitive reserve, brain reserve, and brain maintenance. Alzheimers Dement.

[40] Cheng S-T (2016). Cognitive Reserve and the Prevention of Dementia: the role of physical and cognitive activities. Curr Psychiatry Rep.

[41] Staff RT, Hogan MJ, Williams DS, Whalley LJ (2018). Intellectual engagement and cognitive ability in later life (the use it or lose it conjecture): longitudinal, prospective study. BMJ.

[42] Kuiper JS, Zuidersma M, Oude Voshaar RC, Zuidema SU, van den Heuvel ER, Stolk RP, Smidt N (2015). Social relationships and risk of dementia: a systematic review and meta-analysis of longitudinal cohort studies. Ageing Res Rev.

[43] Evans IEM, Martyr A, Collins R, Brayne C, Clare L (2019). Social isolation and cognitive function in later life: a systematic review and Meta-analysis. J Alzheimers Dis.

[44] Petersen RC, Lopez O, Armstrong MJ, Getchius TSD, Ganguli M, Gloss D, Gronseth GS, Marson D, Pringsheim T, Day GS (2018). Practice guideline update summary: mild cognitive impairment: report of the Guideline Development, Dissemination, and implementation Subcommittee of the American Academy of Neurology. Neurology.

[45] Yang L, Martikainen P, Silventoinen K, Konttinen H (2016). Association of socioeconomic status and cognitive functioning change among elderly Chinese people. Age Ageing.

[46] Karlamangla AS, Miller-Martinez D, Aneshensel CS, Seeman TE, Wight RG, Chodosh J (2009). Trajectories of cognitive function in late life in the United States: demographic and socioeconomic predictors. Am J Epidemiol.

[47] Pettigrew C, Soldan A (2019). Defining Cognitive Reserve and implications for cognitive aging. Curr Neurol Neurosci Rep.

