# Supplementary material for: Social support and cognitive activity and their associations with incident cognitive impairment in cognitively normal older adults
Source: BMC Geriatr. 2024 Jan 9;24:38. doi: 10.1186/s12877-024-04655-5 (PMC10775559; doi:10.1186/s12877-024-04655-5)
Supplement: Supplementary file 1 — Supplementary Material 1 [file 12877_2024_4655_MOESM1_ESM.docx]

**eTable 1. The coding of the variables used in the study**

| Variable | Coding |
| --- | --- |
| **Demographic characteristics** |  |
| Sex | 1=male, 2=female |
| Baseline age | 65~116 |
| Education | 1= less than primary school, 2= primary school, 3= junior high school and over |
| Occupation | 1= occupations of lower economic status(commercial, service or industrial worker/ self-employed/ agriculture, forestry, animal husbandry or fishery worker/ houseworker/ never worked)， 2= occupations of higher economic status(professional and technical personnel/ governmental, institutional or managerial personnel/ military personnel) |
| Household income | 1=less than 12000 CNY, 2=12000 CNY or more |
| Residence | 1=urban, 2=town, 3=rural |
| Marital status | 1=married, 2=divorced/ widowed/ never married |
| Living with | 1=living with family members, 2=alone, 3=at nursing home |
| **Healthy living lifestyles** |  |
| Smoking | 0=yes; 1=no |
| Drinking | 0=yes; 1=no |
| Physical activity score |  |
| Total score | 0~8 |
| Housework | 0=never, 1=sometimes, 2=usually |
| Gardening | 0=never, 1=sometimes, 2=usually |
| Personal outdoor activities | 0=never, 1=sometimes, 2=usually |
| Raising domestic animals | 0=never, 1=sometimes, 2=usually |
| Diet score | 0~12 (The food groups included whole/refined grains, fresh vegetables, mushrooms/algae, fresh fruits, vegetable/animal oils, fish, soybeans, garlic, nuts, tea, and sugar. We gave a score of 0, 0.5, or 1 for most food items and 0 or 1 for the three items related to staple foods and primary cooking oils. The higher scores indicated better adherence to the cMIND diet.) |
| **Health Measures** |  |
| ADLs | 14~42 |
| Number of chronic diseases | 0~21 (including hypertension, diabetes, heart disease, stroke and cerebrovascular disease, bronchitis/emphysema/ pneumonia/asthma, tuberculosis, cataract, glaucoma, cancer, prostate tumor, gastric or duodenal ulcer, parkinson, bedsore, arthritis, epilepsy, cholecystitis/gallstone, blood disease, chronic nephritis, galactophore disease, uterine tumor, hepatitis) |
| **Social support** |  |
| Total score | 0~15 |
| What social security and commercial insurance do you currently have? | 0=no, 1=yes |
| What social services for the elderly are available in your community? | 0=no, 1=yes |
| Who do you chat with the most? | 0=nobody, 1=social worker or housekeeper, 2= child/friend/relative, 3=spouse |
| If you have something on your mind or an idea, who do you talk to first? | 0=nobody, 1=social worker or housekeeper, 2= child/friend/relative, 3=spouse |
| Who do you ask first for help when you have problems or difficulties? | 0=nobody, 1=social worker or housekeeper, 2= child/friend/relative, 3=spouse |
| Who takes care of you when you are sick? | 0=nobody, 1=social worker or housekeeper, 2= child/friend/relative, 3=spouse |
| Do you receive money from the children? | 0=no, 1=yes |
| **Cognitive activities** |  |
| Total score | 0~8 |
| Reading/Surf the Internet | 0=never, 1=sometimes, 2=usually |
| Play cards/mah-jong | 0=never, 1=sometimes, 2=usually |
| Watching TV/listening to the radio | 0=never, 1=sometimes, 2=usually |
| Organized social activities | 0=never, 1=sometimes, 2=usually |
| **MMSE** |  |
| Total score | 0~30 |

**eTable 2. Definitions of the components of the four-way decomposition with relevance to the study^1, 2^**

| Components | Counterfactual definition | Explanation | Contextual definition |
| --- | --- | --- | --- |
| Total effect (TE) | *Y_a_ − Y_a∗_* | Total effect of *A* (changing from *a^∗^* to *a*) on the outcome *Y* | What is the risk of cognitive impairment among those with a high level of social support in comparison with those with low level of social support? |
| Controlled direct effect (CDE) | *Y_am∗_ − Y_a∗m∗_* | Effect of exposure *A* (changing from *a^∗^* to *a*) on the outcome *Y* , with the mediator *M* fixed at level *m*. The CDE defines the component of TE that is due neither to interaction nor mediation. | What is the risk of cognitive impairment among those with a high level of social support in comparison with those with a low level of social support, if everyone attained the same given level of cognitive activities? |
| Reference interaction (INTref ) | *(Y_am_ − Y_am∗_ − Y_a∗m_ + Y_a∗m∗_)(M_a∗_)* | An additive interaction that only operates if the mediator is present when the exposure *A* is *a*. The INTref defines the component of TE that is due to interaction only (but not mediation). | What is the combined risk of cognitive impairment among those with a high level of social support and a high level of cognitive activities, if social support does not have an effect on cognitive activities? |
| Mediated interaction (INTmed) | *(Y_am_−Y_am∗_ −Y_a∗m_+Y_a∗m∗_)(M_a_−M_a∗_)* | An additive interaction that operates if exposure A has an effect on the mediator M (*Ma* − *Ma^∗^≠*0). The INTmed defines the component of TE that is due to both interaction and mediation. | What is the combined risk of cognitive impairment among those with high level of social support and high level of cognitive activities, if social support has an effect on cognitive activities? |
| Pure indirect effect (PIE) | *(Y_a∗m_ − Y_a∗m∗_)(M_a_ − M_a∗_)* | The effect of the mediator (changing from m^∗^ to m) on the outcome Y when exposure A is a, multiplied by the effect of the exposure A (changing from a^∗^ to a) on the mediator M. The PIE defines the component of TE that is due to mediation only (but not interaction) | What is the risk of cognitive impairment among those with a high level of social support in comparison with those with a low level of social support, if social support has an effect on cognitive activities? |

**eReference:**

e1. Discacciati A, Bellavia A, Lee JJ, Mazumdar M, Valeri L. Med4way: a Stata command to investigate mediating and interactive mechanisms using the four-way effect decomposition. Int J Epidemiol 2018:15-20.

e2. Yu B, Feng C, Yang X, et al. Roles of Social Capital in the Association Between Internalized Homophobia and Condomless Sex Among Men Who Have Sex With Men in Southwest China: A Four-Way Decomposition. Int J Public Health 2023;68:1605202.

**eTable 3. Subgroup analyses: the effects of social support on cognitive impairment due to mediation and interaction with cognitive activity**

| Variable | Cognitive impairment  case/person, No. | Total effect | CDE | |  | INTref | |  | INTmed | |  | PIE | |
| --- | --- | --- | --- | --- | --- | --- | --- | --- | --- | --- | --- | --- | --- |
|  |  | Excess RR (95%CI) | Excess RR (95%CI) | % |  | Excess RR (95%CI) | % |  | Excess RR (95%CI) | % |  | Excess RR (95%CI) | % |
| **Subgroup analyses** | |  |  |  |  |  |  |  |  |  |  |  |  |
| **Age group** | |  |  |  |  |  |  |  |  |  |  |  |  |
| 65~79 | 406/3493 | -0.140(-0.307,0.027) | -0.163(-0.335,0.009) | 116.4 |  | 0.030(-0.025,0.085) | -21.7 |  | 0.011(-0.009,0.031) | -7.7 |  | -0.018(-0.037,0.001) | 13.0 |
| ≥80 | 1268/5901 | -0.066(-0.125,-0.008)* | -0.060(-0.119,-0.002)* | 90.9 |  | 0.003(-0.014,0.02) | -4.6 |  | -0.001(-0.004,0.003) | 0.8 |  | -0.009(-0.015,-0.002)* | 12.9 |
| **Sex** |  |  |  |  |  |  |  |  |  |  |  |  |  |
| Male | 628/4570 | -0.126(-0.264,0.011) | -0.109(-0.255,0.037) | 86.1 |  | 0.003(-0.019,0.025) | -2.2 |  | 0.001(-0.016,0.019) | -1.1 |  | -0.022(-0.038,-0.005)* | 17.2 |
| Female | 1046/4824 | -0.048(-0.081,-0.015)* | -0.043(-0.077,-0.01)* | 90.4 |  | -0.001(-0.01,0.008) | 2.4 |  | 0.0004(-0.001,0.001) | -0.3 |  | -0.004(-0.006,-0.001)* | 7.4 |
| **Education** |  |  |  |  |  |  |  |  |  |  |  |  |  |
| Less than primary school | 1096/5355 | -0.049(-0.080,-0.017)* | -0.053(-0.085,-0.021)* | 109.4 |  | 0.009(-0.005,0.022) | -18.2 |  | -0.001(-0.002,0.0005) | 1.2 |  | -0.004(-0.006,-0.001)* | 7.6 |
| Primary school and over | 578/4039 | -0.117(-0.265,0.031) | -0.081(-0.234,0.071) | 69.5 |  | -0.021(-0.054,0.012) | 17.6 |  | 0.013(-0.008,0.033) | -10.9 |  | -0.028(-0.047,-0.009)* | 23.8 |
| **Household income** |  |  |  |  |  |  |  |  |  |  |  |  |  |
| Less than 12000 CNY | 868/4795 | -0.089(-0.182,0.004) | -0.095(-0.188,-0.002)* | 106.6 |  | 0.021(-0.006,0.048) | -23.2 |  | -0.005(-0.012,0.002) | 5.6 |  | -0.010(-0.018,-0.002)* | 11.0 |
| 12000 CNY or more | 806/4599 | -0.166(-0.266,-0.066)* | -0.169(-0.275,-0.062)* | 101.8 |  | 0.013(-0.002,0.028) | -8.1 |  | 0.010(-0.002,0.021) | -5.8 |  | -0.020(-0.033,-0.007)* | 12.0 |
| **ADLs** |  |  |  |  |  |  |  |  |  |  |  |  |  |
| 14 points | 527/4077 | -0.119(-0.276,0.038) | -0.157(-0.316,0.002) | 132.0 |  | 0.041(-0.009,0.091) | -34.6 |  | 0.014(-0.004,0.033) | -12.1 |  | -0.017(-0.034,-0.001)* | 14.7 |
| 15~18 points | 526/2428 | -0.084(-0.168,-0.0005)* | -0.073(-0.158,0.013) | 86.5 |  | -0.002(-0.013,0.010) | 1.8 |  | 0.001(-0.004,0.005) | -1.0 |  | -0.011(-0.020,-0.002)* | 12.8 |
| >18 points | 621/2889 | -0.063(-0.152,0.025) | -0.015(-0.159,0.128) | 24.0 |  | -0.041(-0.127,0.044) | 65.4 |  | -0.002(-0.007,0.003) | 3.5 |  | -0.005(-0.011,0.002) | 7.1 |

Abbreviations: CDE = controlled direct effect; INTref = reference interaction effect; INTmed = mediated interaction effect; PIE = pure indirect effect; RR = risk ratio; CA = cognitive activity; ADL = activities of daily living.

Note: The following adjusted variables were included in each model: age, sex, education, residence, occupation, household income, living pattern, smoking, drinking, physical activity score, diet score, ADL, and number of chronic diseases. * *P*<0.05.

**eTable 4.** **The effects of social support on cognitive impairment due to mediation and interaction with cognitive activities**

| Variable | Cognitive impairment  case/person, No. | Total effect | CDE | |  | INTref | |  | INTmed | |  | PIE | |
| --- | --- | --- | --- | --- | --- | --- | --- | --- | --- | --- | --- | --- | --- |
|  |  | Excess RR (95%CI) | Excess RR (95%CI) | % |  | Excess RR (95%CI) | % |  | Excess RR (95%CI) | % |  | Excess RR (95%CI) | % |
| **Excluding death in 2011 ^a^** | |  |  |  |  |  |  |  |  |  |  |  |  |
|  | 1674/6698 | -0.130(-0.198,-0.061)* | -0.117(-0.186,-0.047)* | 90.0 |  | 0.001(-0.002,0.005) | -1.1 |  | 0.0005(-0.005,0.006) | -0.4 |  | -0.015(-0.022,-0.007)* | 11.4 |
| **Individual chronic diseases as confounders ^b^** | |  |  |  |  |  |  |  |  |  |  |  |  |
|  | 1674/9394 | -0.124(-0.192,-0.057)* | -0.111(-0.18,-0.043)* | 89.4 |  | 0.001(-0.004,0.005) | -0.6 |  | 0.0002(-0.006,0.006) | -0.1 |  | -0.014(-0.021,-0.007)* | 11.4 |
| **Using cognitive activity without participating in social activities as a mediator ^a^** | |  |  |  |  |  |  |  |  |  |  |  |  |
|  | 1674/9394 | -0.125(-0.192,-0.057)* | -0.115(-0.183,-0.047)* | 92.1 |  | 0.002(-0.009,0.014) | -1.7 |  | -0.001(-0.005,0.004) | 0.4 |  | -0.011(-0.018,-0.005)* | 9.2 |
| **Participants aged 60 years and older ^a^** | |  |  |  |  |  |  |  |  |  |  |  |  |
|  | 1680/9533 | -0.127(-0.194,-0.059)* | -0.114(-0.182,-0.045)* | 89.7 |  | 0.001(-0.003,0.005) | -0.6 |  | 0.0002(-0.006,0.006) | -0.2 |  | -0.014(-0.021,-0.007)* | 11.1 |

Abbreviations: CDE, controlled direct effect; INTref, reference interaction effect; INTmed, mediated interaction effect; PIE, pure indirect effect; RR, risk ratio; CA, cognitive activities.

Noto: a, the following adjusted variables were included in each model: age, sex, education, residence, occupation, household income, living pattern, smoking, drinking, physical activity score, diet score, ADL, and number of chronic diseases. b, the model was adjusted age, sex, education, residence, occupation, household income, living pattern, smoking, drinking, physical activity score, diet score, ADL, **hypertension, and diabetes**. * *P*<0.05.

**eTable 5.** Demographic characteristics of participants who lost follow-up (including participants with missing major variables) and participants in study

|  |  | Overall | Participants who lost follow-up | Participants in study |
| --- | --- | --- | --- | --- |
| N (%) |  | 11148 | 1754 | 9394 |
| Sex (%) | Male | 5379 (48.3) | 809 (46.1) | 4570 (48.6) |
|  | Female | 5769 (51.7) | 945 (53.9) | 4824 (51.4) |
| Baseline age (mean [SD]) | | 83.75 (10.86) | 84.84 (11.21) | 83.54 (10.78) |
| Education (%) | Less than primary school | 6305 (56.6) | 950 (54.2) | 5355 (57.0) |
|  | Primary school | 3550 (31.8) | 558 (31.8) | 2992 (31.9) |
|  | Junior high school and over | 1293 (11.6) | 246 (14.0) | 1047 (11.1) |
| Occupation (%)^a^ | Manual laborers | 10106 (90.7) | 1540 (87.8) | 8566 (91.2) |
|  | Non-manual laborers | 1042 (9.3) | 214 (12.2) | 828 (8.8) |
| Household income (%)^b^ | Less than 12000 CNY | 5425 (48.7) | 630 (35.9) | 4795 (51.0) |
|  | 12000 CNY or more | 5723 (51.3) | 1124 (64.1) | 4599 (49.0) |
| Residence (%) | Urban | 2328 (20.9) | 711 (40.5) | 1617 (17.2) |
|  | Town | 2241 (20.1) | 294 (16.8) | 1947 (20.7) |
|  | Rural | 6579 (59.0) | 749 (42.7) | 5830 (62.1) |
| Marital status (%) | Married | 4485 (40.2) | 606 (34.5) | 3879 (41.3) |
|  | Divorced/ widowed/ never married | 6663 (59.8) | 1148 (65.5) | 5515 (58.7) |
| Living with (%) | Living with family members | 9109 (81.7) | 1370 (78.1) | 7739 (82.4) |
|  | Alone | 1873 (16.8) | 338 (19.3) | 1535 (16.3) |
|  | At nursing home | 166 (1.5) | 46 (2.6) | 120 (1.3) |
| Smoking (%) | No | 8927 (80.1) | 1476 (84.2) | 7451 (79.3) |
|  | Yes | 2221 (19.9) | 278 (15.8) | 1943 (20.7) |
| Drinking (%) | No | 9024 (80.9) | 1500 (85.5) | 7524 (80.1) |
|  | Yes | 2124 (19.1) | 254 (14.5) | 1870 (19.9) |
| Physical activity score (mean [SD]) | | 3.11 (2.02) | 2.93 (2.00) | 3.15 (2.02) |
| Diet score (mean [SD]) | | 4.95 (1.60) | 5.31 (1.65) | 4.88 (1.58) |
| ADLs (mean [SD]) | | 18.10 (5.64) | 18.90 (5.94) | 17.95 (5.57) |
| Chronic disease No. (mean [SD]) | | 1.01 (1.21) | 1.17 (1.37) | 0.98 (1.17) |
| Cognitive activity score at baseline (mean [SD]) | | 2.13 (1.64) | 2.30 (1.70) | 2.10 (1.63) |
| Social support score at baseline (mean [SD]) | | 10.90 (2.24) | 10.70 (2.31) | 10.94 (2.23) |
| MMSE score at baseline (mean [SD]) | | 26.84 (3.30) | 26.82 (3.35) | 26.85 (3.29) |
